# Assessment of Association Between Venous Occlusion and Infection of Cardiac Implantable Electronic Devices

**DOI:** 10.1177/00033197211038376

**Published:** 2021-08-06

**Authors:** Andreas Keyser, Carsten Jungbauer, Janine Rennert, Birgit Linnemann, Christof Schmid, Simon Schopka

**Affiliations:** 1 Department of Cardiothoracic Surgery, 210421 University Medical Center, Regensburg, Germany; 2 Department of Cardiology, 210421 University Medical Center, Regensburg, Germany; 3 Department of Radiology, 210421 University Medical Center, Regensburg, Germany; 4 Department of Vascular Surgery and Angiology, 210421 University Medical Center, Regensburg, Germany

**Keywords:** cardiac implantable electronic devices, endocardium, infection, venography, venous patency, venous occlusion

## Abstract

The increasing number of patients treated with cardiac implantable electronic devices (CIEDs) and indications for complex pacing requires system revisions. Currently, data on venous patency in repeat CIED surgery involving lead (re)placement or extraction are largely missing. This study aimed to assess venous patency and risk factors in patients referred for repeat CIED lead surgery, emphasizing CIED infection. All consecutive patients requiring extraction, exchange, or additional placement of ≥1 CIED leads during reoperative procedures from January 2015 to March 2020 were evaluated in this retrospective study. Venography was performed in 475 patients. Venous patency could be assessed in 387 patients (81.5%). CIED infection with venous occlusion was detected in 74 patients compared with venous occlusion without infection in 14 patients (*P* < .05). Concerning venous patency, novel oral anticoagulant medication appeared to be protective (*P* < .05; odds ratio [OR]: .35). Infection of the CIED appeared to be strongly associated with venous occlusion (OR: 16.0). The sensitivity was only 64.15%, but the specificity was 96.1%. Number of leads involved and previous CIED procedures were not associated with venous occlusion. In conclusion, in patients with CIED, venous occlusion was strongly associated with device infection, but not with the number of leads or previous CIED procedures.

## Introduction

The increasing number of patients treated with cardiac implantable electronic devices (CIEDs) and increasing indications for complex pacing heightened the need for system revisions due to lead failure as well as upgrade and extraction procedures.^1,2^ In repeat CIED surgery, including additional lead placement or exchange, ensuring venous patency is essential to plan for these surgical procedures.^3,4^

The prevalence of venous occlusion (VO) in lead extraction has been described.^3,4^ VO is a considerable obstacle for standard lead placement or exchange. To the best of our knowledge, data on venous patency in patients undergoing repeat CIED lead surgery are sparse, in particular with regard to CIED infections. Thus, the aim of this study was to assess venous patency and risk factors in patients referred for repeat CIED lead surgery with special emphasis on CIED infection.

## Methods

### Patient Cohort

This retrospective study collected data from all consecutive patients in need of repeat CIED-related surgery involving lead exchange, removal, or additional placement from January 2015 to March 2020. Patients who underwent battery exchanges and lead revisions within 1 month after an initial surgery were excluded from the analysis.

Definitions

Local infection was defined as infection restricted to the CIED pocket site. Local signs of erythema, warmth, fluctuance, tenderness, purulent discharge, erosion, and wound dehiscence at the pocket site enabled establishing the diagnosis of local infection. Lead-related infective endocarditis was defined as an infection of the leads, endocardial surface, or cardiac valve leaflets. Vegetation, defined as additional mobile mass on the leads, valves, or endocardium, was visualized in at least 2 echocardiographic planes. The diagnosis of CIED infection was based on the modified Duke criteria.^5^ Infections unrelated to the CIED system were classified as non-CIED infections.

### Digital Subtraction Venography

Digital subtraction venography was an integral part of patient preparation for surgery. The patients with contrast medium hypersensitivity or renal dysfunction were prepared for venography prior to examination and treated accordingly thereafter. Venography was performed on the side of former lead placement. In case of bilateral leads, venography was performed on both sides. Contrast media (10–25 mL, Optiray®, manufactured by Villepinte, France) was injected into the ipsilateral antecubital vein. Images were taken with anteroposterior view. Two heart surgeons, 1 cardiologist, and 1 radiologist, all experienced, reviewed the venograms prior to surgery. Vessel patency was graded as occlusion if there was a clear interruption of contrast flow with extensive new collateral veins. Otherwise, the vessel was considered open. Vessels with partial stenosis and lack of collateral veins were also categorized as open.

### Patient Data

The patients’ data pertaining to the implant side, indication for initial CIED placement, numbers of prior CIED procedures, and indication for repeat procedure were recorded. In addition, heart rhythm and number, types of leads present, mobile masses on echocardiography, and institution where the last CIED procedure was performed were noted. Furthermore, data on age, sex, body mass index (BMI), and left ventricular ejection fraction, as well as a history of hypertension, coronary heart disease, and peripheral artery disease were extracted. Additionally, data on history of cerebral ischemia, diabetes mellitus, renal insufficiency, glomerular filtration rate (GFR) <60 mL/min/1.73 m,^2^ pulmonary disease, history of malignant disease, history of venous thrombosis or pulmonary embolism, smoking and alcohol, dyslipidemia, history of cardiac surgery, and anticoagulant therapy were collected. Swabs for microbiological workup from all generator pockets and leads were obtained during surgery.

The local ethics committee approved the study protocol (No. 17-9-104). All included patients provided written informed consent which was approved by the Institutional Ethics Committee (IRB) and was performed according to the principles expressed in the Declaration of Helsinki.

### Statistics

Continuous variables were expressed as median ±25th and 75th percentile. Categorical variables were represented as counts and percentages of the respective data. The continuous variables were analyzed by the Mann–Whitney Rank sum test. Categorical variables were compared using the χ^2^ test. Variables analyzed between the patients with occluded and patent venous access were introduced to the multivariate model to select independent predictive factors for venous occlusion. Analyses were 2-sided, and differences with a *P* < .05 were considered significant. Analyses were performed using SigmaPlot 13.0 (Systat Software GmbH., Erkrath, Germany).

## Results

### Patient Data

A total of 483 patients were treated during the observation period. Venography could be obtained in 475 patients (the prevalence of venography was 98.3%), which served as a basis for consecutive statistical analyses.

In the whole patient cohort, the mean age was 65 ± 14 years, and 357 patients were male. The BMI was 28.3 ± 5.6 kg/m^2^. The left ventricular ejection fraction was 40.8 ± 16.7% (range 10–75%).

The CIEDs implanted were cardioverter defibrillators in 286 patients (60.2%), pacemakers in 187 patients (39.4%), and cardiac contractility modulation devices in 2 patients (.4%). The indications for pacemaker and CIED implantations are listed in Table 1. Previous CIED surgery had been performed in an external institution on 298 patients (referral rate 63%). Most of the CIEDs had been implanted on the left side (71.2%). The predominant access was the left subclavian vein with subcutaneous generator positioning.

**Table 1.** Pacemaker and Cardioverter-Defibrillator Indication and Implanted Devices.

Pacemaker	Number of patients
AVB I and AVB II	4
AVB II Mobitz	11
AVB II Wenckebach	5
CAVB	76
SSS	46
TBS	26
Carotid sinus syndrome	4
Bradyarrhythmia	8
ICD-indication (multiple indications possible)	
DCM	131
End-stage CAD	139
HOCM	9
Brugada syndrome	5
Idiopathic VF	13
Others	10
Device	
AAI	1
VVI	31
VDD	5
DDD	148
CRT-P	2
ICD VVI	130
ICD DDD	87
CRT-D	69
CCM	2

Abbreviations: AVB, atrioventricular block; CAVB, complete atrioventricular block; SSS, sick sinus syndrome; TBS, tachycardia bradycardia syndrome; ICD, implantable cardioverter-defibrillator; DCM, dilative cardiomyopathy; CAD, coronary artery disease; HOCM, hypertrophic obstructive cardiomyopathy; VF, ventricular fibrillation; CRT-P, cardiac resynchronization therapy pacemaker; CRT-D, cardiac resynchronization therapy defibrillator; CCM, cardiac contractility modulation; AAI, atrial inhibiting pacer; VVI, ventricular inhibiting pacer; VDD, atrial synchronized ventricular pacer; DDD, atrioventricular universal pacer; ICD VVI, implantable cardioverter-defibrillator/ventricular inhibiting pacer; ICD DDD, implantable cardioverter-defibrillator/atrioventricular universal pacer.

More than one previous CIED procedure was noted in 288 patients (60.6%). Previous CIED procedures involving lead surgery more than once were identified in 137 patients (28.8%). The total number of leads per patient ranged from 1 to 5 with a dwell time of 1 to 355 months. The data are summarized in Table 2.

**Table 2.** Details of implanted devices.

Implantation side	Number of patients
Right	127
Left	338
Both	10
Venous access^†^	
Cephalic cut down	84
Subclavian puncture	391
Generator position	
Submuscular	204
Subcutaneous	271
Number of prior procedures	
1	247
2	114
3	69
4	32
5	7
6	4
7	1
10	1
Number of prior procedures involving leads	
1	338
2	101
3	31
4	5
Number of leads per patient	
1	144
2	238
3	74
4	17
5	2
Total number of leads	920
Dwell time of leads (months)	79.5 ± 70.0 range 1–355
2 different dwell times	88
3 different dwell times	8

^†^In case of cephalic cut down and subclavian puncture, subclavian puncture as access was chosen.

During our surgical intervention, 478 leads were removed (1.02 leads/patient), and lead exchange was necessary in 210 patients (.44/patient). System upgrades were performed in 97 patients (.20/patient). Procedural data are summarized in Table 3.

**Table 3.** Procedure for repeat CIED surgery involving leads.

Upgrade	
DDD → ICD DDD	9
DDD → CRT-D	14
ICD VVI → ICD DDD	4
ICD VVI → CRT-D	23
ICD DDD → CRT-D	35
Others	12
Total	97
Lead exchange (multiple lead exchange possible)	
RA P/S	41
RV P/S	52
RV ICD	103
LV P/S	14
Total	210
Leads removed	
Simple traction	156
Laser lead extraction	210
Polypropylene sheath extraction	112
Total	478 (25 myocardial perforation)
Leads explanted	478
Leads remaining	442

Abbreviations: CIED, cardiac implantable electronic device; →, upgrade from one system to other; DDD, atrioventricular universal pacer; ICD DDD, implantable cardioverter-defibrillator/atrioventricular universal pacer; ICD VVI, implantable cardioverter-defibrillator/ventricular inhibiting pacer; ICD DDD, implantable cardioverter-defibrillator/atrioventricular universal pacer; CRT-D, cardiac resynchronization therapy defibrillator; RA, right atrial; P/S, pace-sense; RV, right ventricular; LV, left ventricular.

### Infection

Diagnosis of CIED infection according to the modified Duke lead criteria^5^ could be established in 114 patients (24%) prior to surgery. Only 67 patients (58.8%; overall, 14.1%) of these 114 also presented with unmistakable signs of a pocket infection. A lead-associated infective endocarditis was detected in 47 patients (41.2%; overall, 9.9%).

### Findings of Digital Subtraction Venography

Venous patency was confirmed in 387 patients (81.5%). Among these, 40 patients (10.3%) were treated for obvious infection of CIED. Venous occlusion was found in 88 patients (18.5%) with subclavian vein occlusion in 37 patients (7.8%), brachiocephalic vein occlusion in 21 patients (4.4%), occlusion of the subclavian and brachiocephalic veins in 27 patients (5.7%), and occlusion of the superior caval vein in 3 patients (.6%).

Patients (n=347) without infection demonstrated patent veins, whereas 14 non-infected patients presented venous occlusion (*P* < .05). In 2 patients, occlusion of a prosthetic replacement of the left brachiocephalic vein after cardiac surgery was documented, and 1 patient presented with occlusion of the left brachiocephalic vein related to an aortic arch aneurysm. In case of CIED infection, venous occlusion was more frequent (74 patients) compared with venous patency (40 patients) (*P* < .05). The odds ratio for venous occlusion in patients with CIED infection was calculated to 16.0 (95% confidence interval [CI]: 9.54–26.81) and the relative risk to 11.38 (95% CI 7.03–18.82). The sensitivity for venous occlusion to predict CIED infection was only 64.2%, whereas specificity was 96.1%.

### Risk Factors

#### Univariate analysis

In univariate analysis, venous occlusion was significantly associated with CIED infection and non-CIED infections compared with non-infected CIED (each *P* < .05). Right-sided implantation of CIED leads had a significantly higher rate of venous occlusion compared with left-sided implantation (*P* < .05). Occlusion of the upper veins significantly increased with the number of CIED procedures and number of indwelling leads (*P* < .05) as well as with a history of malignant disease (*P* < .05) (Table 4). There was no difference in venous occlusion rate between pacemakers and ICDs (*P* = .57, OR: .87, 95% CI: .54–1.4).

**Table 4.** Univariate Analysis.

Category	V. subclavia patent	V. subclavia occluded	p
Patients	387 (81.5%)	88 (18.5%)	—
Body mass index (kg/m^2^)	27.4 (24.3/31.1)	27.8 (24.7/31.3)	.545
Age	66 (56/77)	68 (60.3/76.8)	.071
Female	102 (28.3%)	16 (14.0%)	.109
Right-sided implantation	**101 (27.9%)**	**34 (29.8%)**	**<.05**
Subclavian puncture	320 (67.4%)	110 (23.2%)	.317
Left ventricular ejection fraction	38 (25/55)	45.5 (30/59.8)	.052
Hypertension	220 (60.9%)	53 (46.5%)	.563
Coronary artery disease	184 (51.0%)	47 (41.2%)	.321
Peripheral vascular disease	68 (18.8%)	22 (19.3%)	.108
History of cerebral ischemia	45 (12.5%)	8 (7.0%)	.495
Diabetes mellitus	189 (52.4%)	36 (31.6%)	.179
Renal insufficiency	182 (50.4%)	34 (29.8%)	.154
Dialysis	18 (5.0%)	5 (4.4%)	.684
Pulmonary disease	104 (28.8%)	32 (28.1%)	.100
History of malignant disease	**35 (9.7%)**	**18 (15.8%)**	**<.05**
History of thrombosis/pulmonary embolism	26 (7.2%)	9 (7.9%)	.255
Smoking	148 (40.1%)	36 (31.6%)	.643
Dyslipidemia	108 (29.9%)	24 (21.1%)	.905
Alcohol consumption >20g daily	62 (17.2%)	17 (14.9%)	.453
Previous cardiac surgery	118 (32.7%)	23 (20.2%)	.420
Atrial fibrillation	153 (42.4%)	45 (39.5%)	.061
Platelet inhibitor treatment	137 (38.0%)	22 (19.3%)	.082
Phenprocoumon treatment	129 (35.7%)	36 (31.6%)	.178
Novel oral anticoagulation	91 (25.2%)	19 (16.7%)	.699
Number of CIED procedures	**1 (1/2)**	**2 (1/3)**	**< .05**
Number of CIED procedures involving leads	1 (1/2)	1 (1/2)	.105
Number of leads	**2 (1/2)**	**2 (2/3)**	**< .05**
Implantable cardioverter-defibrillator	237 (65.7%)	48 (42.1%)	.247
Infected CIED	**40 (11.1%)**	**74 (65%)**	**< .05**
Non-CIED infection	**33 (9.1%)**	**16 (14%)**	**< .05**

Abbreviations: V, subclavia; subclavian vein, CIED; cardiac implantable electronic device. The significance for the use of bold is p < 0.05.

#### Multivariate logistic regression

The major risk factor for CIED infection in multivariate regression was venous occlusion (*P* < .001; odds ratio [OR]: 76.09; 95% CI: 32.77–176.65). Coronary heart disease and a history of heart surgery were also risk factors for venous occlusion, but to a lesser extent (*P* = .030; OR: 2.30; 95% CI: 1.08–4.89 and *P* = .046; OR: 1.99; 95% CI: 1.01–3.95, respectively). Novel oral anticoagulant medications appeared to be protective with regard to venous patency (*P* = .021, OR: .326, 95% CI: .13–.85).

Neither the number of prior CIED procedures (*P* = .482; OR: .86, 95% CI: .57–1.30), nor the number of previous CIED procedures involving leads (*P* = .133, OR: .54, 95% CI: .25–1.20) was predictive for venous occlusion in the multivariate logistic regression. This was also true for the number of leads involved (*P* = .963, OR: 1.01, 95% CI: .65–1.56) (Table 5).

**Table 5.** Multivariate analysis.

Individual variable	Coefficient	Standard error	Wald statistic	p	Odds ratio	5% conf. lower	95% conf. upper
Constant	−4.057	1.464	7.677	0.006	0.0173	0.000981	0.305
Body mass index	0.0323	0.0305	1.125	0.289	1.033	0.973	1.096
Age	0.0144	0.0122	1.388	0.239	1.015	0.990	1.039
Female	−0.728	0.401	3.296	0.069	0.483	0.220	1.060
Right-sided implantation	0.346	0.352	0.964	0.326	1.413	0.708	2.820
Subclavian puncture	0.162	0.455	0.127	0.721	1.176	0.482	2.868
Left ventricular ejection fraction	0.00954	0.0123	0.606	0.436	1.010	0.986	1.034
Hypertension	−0.180	0.346	0.270	0.603	0.835	0.424	1.646
Coronary artery disease	**0.833**	**0.385**	**4.696**	**0.030**	**2.301**	**1.083**	**4.889**
Peripheral vascular disease	−0.528	0.472	1.249	0.264	0.590	0.234	1.489
History of cerebral ischemia	−0.620	0.599	1.071	.301	.538	.166	1.741
Diabetes mellitus	−0.757	0.391	3.749	0.053	0.469	0.218	1.009
Renal insufficiency	0.419	0.364	1.325	0.250	1.521	0.745	3.104
Dialysis	−0.592	0.897	0.436	0.509	0.553	0.0953	3.208
Pulmonary disease	0.188	0.372	0.254	0.614	1.206	0.581	2.503
History of malignant disease	0.0748	0.499	0.0225	0.881	1.078	0.405	2.864
History of thrombosis/pulmonary embolism	0.299	0.573	0.273	0.601	1.349	0.439	4.145
Smoking	0.148	0.364	0.165	0.685	1.159	0.568	2.366
Dyslipidemia	0.373	0.366	1.039	0.308	1.452	0.709	2.975
Alcohol consumption >20g daily	−0.821	0.440	3.474	0.062	0.440	0.186	1.043
Previous cardiac surgery	**0.693**	**0.347**	**3.973**	**0.046**	**1.999**	**1.012**	**3.950**
Atrial fibrillation	0.295	0.377	0.611	0.434	1.343	0.641	2.815
Platelet inhibitor treatment	−0.352	0.398	0.782	0.376	0.703	0.323	1.534
Phenprocoumon treatment	−0.0370	0.398	0.00867	0.926	0.964	0.442	2.101
Novel oral anticoagulation	−**1.120**	**0.486**	**5.303**	**0.021**	**0.326**	**0.126**	**0.846**
Number of CIED procedures	−0.147	0.209	0.493	0.482	0.864	0.574	1.300
Number of CIED procedures involving leads	−0.607	0.404	2.263	0.133	0.545	0.247	1.202
Number of leads	0.0101	0.221	0.00210	0.963	1.010	0.656	1.557
Implantable cardioverter-defibrillator	0.466	0.415	1.264	0.261	1.594	0.707	3.594
Infected CIED	**4.332**	**0.430**	**101.599**	**<0.001**	**76.086**	**32.771**	**176.653**
Non-CIED infection	−.424	0.569	0.555	0.456	0.654	0.215	1.996

Abbreviations: CIED, cardiac implantable electronic device. The significance for the use of bold is p < 0.05.

## Discussion

This present study evaluated venous patency and risk factors in 475 consecutive patients referred for repeat CIED lead procedure. Venous occlusion was found in 18% of the patients and was significantly correlated with device infection. In addition, venous occlusion was seen less often in patients on novel oral anticoagulants.

The true incidence of venous occlusion in patients with CIEDs remains unclear because of inconsistent findings. In 1976, Stoney et al^6^ identified a venous occlusion rate of 22% on the side of transvenous lead placement in 32 patients. Forty years later, Sohal et al reported a venous occlusion rate of 21.5% in 242 patients, and Li et al also reported a rate of 25.7% in 202 patients.^3,4^ A study of 105 patients scheduled for implantable cardioverter-defibrillator (ICD) generator exchange found a venous occlusion rate of only 9%.^7^ In an autopsy series of 78 patients, no venous occlusion was seen. However, venous thrombi along indwelling leads (dwell time 4.0±3.3 years) were present in 48% of the patients.^8^ Multiple CIED procedures, CIED procedures with lead involvement and consequently intra-individual different dwell times, and finally the lead load of venous vessels were considered to bias the effect of dwell times on venous occlusion rate and therefore were not assessed in the present study.

In patients scheduled for repeat CIED lead surgery, venous occlusion is often symptomless due to the development of collateral circulation. However, repeat surgery is much more difficult and requires more advanced tools in this patient group.^4^ Therefore, we considered assessment of venous patency in repeat CIED lead surgery, particularly to plan for this surgical procedure.^3,4^ Venography provides excellent visualization of venous anatomy, in contrast to ultrasound that may fail to detect short or central venous occlusions. However, digital subtraction phlebography can rarely definitively clarify the cause of subclavian vein occlusion, whether it is a blood clot or tissue scar or both. Although venography requires the use of iodinated contrast agent, which may cause an allergic reaction, nephrotoxicity, or phlebitis that could worsen pre-existing thrombosis, we did not observe adverse events in our patients.

Several risk factors for atherosclerosis including hypertension, obesity, diabetes mellitus, dyslipidemia, and nicotine use were assumed to also promote venous occlusion. However, these risk factors are still controversial.^9-11^ We could not confirm the presence of any of the former risk factors. However, we found the presence of coronary artery disease to be significant with an odds ratio of 2.3 (*P* < .05, 95% CI: 1.1–4.9). Age is one of the strongest risk factors for venous thrombosis of the upper extremities in patients with deep venous thrombosis of the lower extremities apart from malignant diseases and genetic risk factors.^12^ Increasing age was also found to be associated with a higher risk of thrombosis in patients with central venous catheters, but not in patients with indwelling leads.^9,13^ In our study, age was not a risk factor. A history of malignant disease was only significant using univariate analysis (upper vein patency 35 patients, 9.7%; upper vein occlusion 18 patients, 15.8%; *P* < .05). Female patients tend to present more frail tissue and presumably smaller vessels than male patients. Linnemann et al^14^ found a majority of female patients matched for sex in a series of 150 patients with upper extremity deep-vein thrombosis. In the present study, female sex was not associated with increased development of venous occlusion, consistent with what has also been reported by van Rooden and Mahmoodi.^9,13^

We also found previous cardiac surgery to be a risk factor for upper venous occlusion. It is well understood that surgical events may have influenced venous patency in this group of patients. Apart from postulated higher age of patients with a history of cardiac surgery, the necessity of central venous catheters during cardiac surgery, injury to the respective vessels, and alterations in the coagulation system are unavoidable during cardiac surgery.

The left-sided subclavian vein is more prone to spontaneous thromboembolism compared with the right-sided one.^15^ Therefore, indwelling leads in the left subclavian vein should probably increase the incidence of thromboembolism and occlusion compared with the right subclavian vein. Interestingly, our study revealed right-sided venous occlusion to be more frequent than left sided occlusion in CIED patients. Anatomical structures may explain this phenomenon. The angle between the subclavian vein and venous truncus is much sharper on the right side compared with the left, where the subclavian vein eases into the left brachiocephalic vein. Shear stress by indwelling leads at the junction of the right subclavian vein may therefore lead to ongoing irritation of the venous wall, thus paving the way to venous stenosis and occlusion. Li et al, who evaluated 202 patients especially referred for lead extraction, found no difference of venous occlusion when compared with the implantation side. However, the total number of implantations of either side was not stated, and might have had an influence on the analysis.^4^

Another parameter which may have had an impact on the rate of venous occlusion is the type of venous access. Subclavian puncture is associated with injury to the vessel wall, and it might become more vulnerable to thrombus formation or vessel occlusion compared with cephalic cut down, a comparably more effective alternative to peripheral access. Moreover, gaining access to the venous system via cephalic cut down does not exclude usage of implantation sheaths. Implantation sheaths, introduced through the cephalic vein, may cause additional damage to the subclavian vein wall. Yet, such a difference related to the access to the venous system has not been substantiated so far. Therefore, it might be assumed that indwelling leads had a higher impact on venous occlusion than the type of access.

Another often presumed covariate of subclavian vein obstruction is the number of leads.^12^ We could confirm this assumption in the univariate analysis, but not in the multivariate analysis. A possible explanation might be that the anatomical structure of the venous walls allows considerable extensions in diameter, unlike that of the arteries’.^14,16^ Blood flow may not be impaired in the presence of several leads. However, with an increasing number of leads, more complex surgery might also be associated with more injury to the venous wall. A number of patients had several different surgical procedures on the same side, which means that they had experienced several repeat surgical procedures with repetitive trauma to the vessel wall.

A special focus of the present study was on the association between CIED infection and venous occlusion. Our findings revealed a strong association between the two. Even if sensitivity of venous occlusion for CIED infection was low, specificity was high and therefore helped to determine the absence of infection. While a link between venous occlusion and infection of CIED was not mentioned by Bongiorni and Boczar, a study by Li et al revealed subclavian venous occlusion in a significant number of patients referred for lead extraction.^4,17,18^ The role of non-CIED infections is less clear. We found this association using univariate analysis only. Infection may cause wide-spread inflammation, activating plasma inflammatory mediators and cell–cell interactions, which promote venous thrombosis and occlusion. On the other hand, primary venous thrombosis followed by secondary infection may occur as well. The absence of blood flow through occluded vessels could lead to a subclinical infection becoming a clinically aberrant infection. Positive microbiological swabs, that is, with evidence of microorganisms from clinically non-infected patients may favor the latter explanation.^19^ However, the discussion will remain speculative, as no data are available on whether pre-existing thrombi or occlusion promote infection or infection promotes development of thrombosis and venous occlusion in this special patient group.

Although atrial fibrillation was not found to be an independent risk factor for upper venous occlusion in the present study, novel oral anticoagulation (NOAC) therapy had a beneficial impact on patency of the upper extremity veins. This was not observed in patients treated with phenprocoumon or platelet inhibitors. In a comparably small study by Li et al,^4^ a correlation between venous patency and anticoagulation was not observed.

### Limitations

This study has several limitations. It is a retrospective study conducted at a single center. There may be a selection bias as more than half of the patients had been referred from other institutions, limiting the generalizability of the findings. Due to reasons mentioned in the discussion section, the impact of dwell times on venous occlusion was not addressed in the present study. The impact of microbiological results on venous patency was also not assessed.

## Conclusion

The prevalence of venous occlusion in CIED patients is high. Venous occlusion in CIED patients was significantly associated with device infection. Venous occlusion was observed less often in patients treated with novel oral anticoagulants. Venography prior to repeat CIED lead surgery is useful to define operative strategies. Multicenter studies with large populations are needed to provide further insights into the development of venous occlusion in CIED patients.
